# Analysis of Woven Fabric Mechanical Properties in the Context of Sustainable Clothing Development Process

**DOI:** 10.3390/polym17152013

**Published:** 2025-07-23

**Authors:** Maja Mahnić Naglić, Slavenka Petrak, Antoneta Tomljenović

**Affiliations:** 1 Department of Clothing Technology, University of Zagreb Faculty of Textile Technology, Prilaz baruna Filipovića 28a, 10000 Zagreb, Croatia; maja.mahnic@ttf.unizg.hr; 2 Department of Materials, Fibers and Textile Testing, University of Zagreb Faculty of Textile Technology, Prilaz baruna Filipovića 28a, 10000 Zagreb, Croatia; antoneta.tomljenovic@ttf.unizg.hr

**Keywords:** woven fabric, mechanical properties, AI technology, 3D simulations, digital prototyping, dynamic deformations

## Abstract

This paper presents research in the field of computer-aided 3D clothing design, focusing on an investigation of three methods for determining the mechanical properties of woven fabrics and their impact on 3D clothing simulations in the context of sustainable apparel development. Five mechanical parameters were analyzed: tensile elongation in the warp and weft directions, shear stiffness, bending stiffness, specific weight, and fabric thickness. These parameters were integrated into the CLO3D CAD software v.2025.0.408, using data obtained via the KES-FB system, the Fabric Kit protocol, and the AI-based tool, SEDDI Textura 2024. Simulations of women’s blouse and trousers were evaluated using dynamic tests and validated by real prototypes measured with the ARAMIS optical 3D system. Results show average differences between digital and real prototype deformation data up to 6% with an 8% standard deviation, confirming the high accuracy of 3D simulations based on the determined mechanical parameters of the real fabric sample. Notably, the AI-based method demonstrated excellent simulation results compared with real garments, highlighting its potential for accessible, sustainable, and scalable fabric digitization. Presented research is entirely in line with the current trends of digitization and sustainability in the textile industry. It contributes to the advancement of efficient digital prototyping workflows and emphasizes the importance of reliable mechanical characterization for predictive garment modeling.

## 1. Introduction

The advancement of computer technologies and CAD systems for 2D/3D garment design has significantly transformed the field of clothing engineering. These innovations have enabled the development of digital 3D prototypes and fit analysis, facilitating the prediction of both the appearance and functionality of garments prior to physical production. The adoption of digital methodologies in garment development significantly contributes to the sustainability of the manufacturing process, addressing the traditional challenges of material waste, time consumption, and financial costs associated with the iterative creation of physical prototypes. Traditionally, garment design and development involve a cyclical process requiring multiple iterations of pattern adjustments and the physical testing of numerous prototypes to achieve the desired garment form and fit. This traditional approach can become especially resource-intensive when dealing with complex designs, as it relies on extensive material usage and manual labor.

In contrast, one of the most notable advantages of CAD systems is their capability to simulate and evaluate the fit of garments directly on 3D virtual human models, without the need for physical samples. Furthermore, these systems allow the assessment of fabric properties in relation to the targeted textile material, thereby facilitating the selection of fabrics based on their physical and mechanical characteristics [1,2]. According to different research and case studies, the implementation of digital prototyping in apparel product development reduces the time of pattern making by 85% and the number of physical samples up to a 60–80% (on average from 3–5 to 1–2 samples), thus significantly reducing material waste, energy consumption and greenhouse gas emissions in the development phase. Saving on sample transport between designers and manufacturers further reduces greenhouse gas emissions and energy costs [3,4,5,6]. The integration of digital prototyping with Industry 4.0 technologies enables resource optimization, sustainability monitoring, and CO_2_ emission reduction through transparent and shortened supply chains, confirming that digital prototyping contributes not only to waste reduction but also to achieving circular economy goals [7,8].

Leading CAD systems, such as Lectra, Optitex, Assyst, CLO3D, and Browzwear, offer comprehensive digital solutions that support the entire garment development process, from 2D pattern construction and 3D body simulation to virtual fitting and integration with marketing channels. These systems are also increasingly employed for virtual fashion shows and online retail visualizations, enabling early-stage product validation and consumer engagement [9,10,11,12,13]. Garment simulation involves physical simulation of joining 2D pattern pieces around a 3D body model, based on algorithms that give virtual garments realistic physical behavior, taking into account forces such as gravity, collisions, and mass [14]. Common simulation techniques are based on elasticity theory, particle-spring systems, and finite element methods (FEM), which are particularly well suited to modeling complex draping and fabric deformation [15,16,17]. The research by Volino and Magnenat Thalmann is particularly significant in developing textile and clothing simulation methods based on elasticity theory and Newtonian dynamics [18,19,20]. Finite element methods are also frequently used to model complex textile deformations and draping [21,22,23].

Given the inherently anisotropic and deformable nature of textiles, simulating the behavior of clothing remains a very complex physical problem. Textiles exhibit significant variations in their technical characteristics, including fiber content, weave structure, yarn density, thickness, and weight, which affect both their manufacturing and wear properties. Mechanical properties are crucial for selecting appropriate materials based on garment design and function. Accurate simulation necessitates the quantification of fabric mechanical properties under low-stress conditions, which are typical during garment handling and wearing [1,2,20]. The main mechanical properties affecting textile behaviour are tensile, bending, shear, and compression properties, along with specific weight and thickness as physical parameters. These characteristics determine the fabric’s response to external forces and are thus essential for accurate virtual prototyping. Since textiles in processing and use are subject to low loads, linearity parameters are key metrics in evaluating processing and performance properties. Thus, tensile values in the linear region along warp and weft directions are most commonly applied for 3D garment simulations. Fabric bending properties depend on composition, construction, and finishing methods, such as dyeing, printing, or surface coatings. Bending stiffness (B), which defines resistance to bending under its weight, is a commonly used parameter for 3D simulations. Shear properties are essential for achieving a proper 3D garment shape, draping, pliability and hand. Shear deformation results from inter-yarn angle changes, and shear stress in the linear range is expressed using shear stiffness. Compression properties in 3D simulation are defined by fabric thickness, measured as the distance between two parallel planes under two different given loads [1,24,25,26].

From the perspective of computer graphics, textile materials in a CAD system for 3D simulation are described as a mesh of polygons with a specified density (i.e., the size of a particular polygon), where each polygon represents a solid surface corresponding to the fabric’s mass. The polygons are interconnected, and the joints between them behave according to the predefined parameters of the fabric’s physical and mechanical properties [1,2]. The quality of a 3D simulation and the ability to predict garment appearance and behaviour heavily depend on the algorithm and number of material parameters each CAD system uses, as well as the method for testing those parameters [27,28]. Tools like the Kawabata Evaluation System (KES-FB), the FAST system, and, more recently, user-friendly devices such as the Fabric Kit and Fabric Testing Utilities integrated into specific CAD software platforms are commonly employed for capturing mechanical parameters in clothing simulations [29,30,31]. Recent research has explored the integration of machine learning algorithms for predicting fabric properties from minimal input parameters, enhancing simulation efficiency. By leveraging extensive datasets, these intelligent systems can correlate physical and mechanical attributes, reducing reliance on complex physical testing [32,33,34].

Numerous comparative studies have assessed the fidelity of various systems and parameter sets in replicating real fabric behavior through 3D simulations [17,29,31,35,36,37,38]. Surface mesh density parameters significantly influence the simulation quality and realistic behavior [14,39,40]. To evaluate 3D simulation results, many studies use complex draping deformation analyses. Kenkara et al. reported that drape parameters vary for each test and that a variability of up to 15% when comparing simulation and real values is acceptable accuracy. Their study was conducted on 20 different fabrics using the Cusick Drape Meter and 3D scanning technology [40]. Thi et al. evaluated drape parameters for six fabrics using V Stitcher [41]. Buyukaslan et al. analyzed drape simulations for five fabrics using the FAST method and the Optitex CAD system. Based on the subjective assessment of the draping parameters and the overall appearance of the simulated samples in comparison with real samples, a satisfactory level of accuracy of the performed 3D simulations was determined, according to Kenkare’s similarity criterion of 15% [40,42]. Rudolf et al. studied the impact of simulation algorithm settings on the simulated skirt model behavior in the Optitex CAD system, based on a comparison between digital and real prototypes, using orthogonal projection areas and length measurements at different positions as metric parameters [43].

To evaluate the computer prototypes and 3D simulation results, various comparisons and evaluation criteria of similarity between the digital and real prototypes are most often used in research [44,45]. Various CAD systems for 3D garment design offer fit analysis tools such as cross-sectioning of a digital garment prototype on a body model, where the amount and distribution of garment ease allowance can be determined from the differences in the circumference values. Analysis of the garment pressure against the body and surface deformations, such as warp or weft elongations, enables fit assessment in relation to the parameters of the mechanical properties of the woven material being simulated [44,46]. Zangue et al. investigated the possibilities of digital prototypes fit analysis, using the example of a men’s jacket and comparison of the results obtained using three different CAD systems for garment design [46]. In their paper, they point out that the assessment of the digital prototypes’ fit is a major challenge that requires a combination of understanding of simulation algorithms and knowledge and experience in garment design and construction. Kim et al. developed an evaluation method based on the comparison of digital and real prototypes of a skirt model using 3D scanning technology for better measurement accuracy. A special contribution of the method is the set of subjective evaluation criteria for the prototype’s appearance and its correlation with objective mechanical parameter values, resulting in a regression equation for evaluating 3D prototypes [47]. Lapkovska and Dabolina also conducted an evaluation study of 3D simulations by comparing computer and simulated prototypes of skirt models, using the parameters of the shape and dimensions of the model cross-sections at different heights as a comparative criterion. They also applied 3D scanning technology to analyze the real prototypes because it is not possible to simply and precisely determine the shape and dimensions of the garment cross-sections using the conventional methods due to the folds of the material [48].

Despite notable advancements in virtual analysis of garment fit, limitations remain, particularly in the objective assessment of digital prototypes during motion. However, validation techniques such as 3D scanning and optical measurement systems, such as those used in the presented study, offer promising approaches to quantitatively evaluating virtual fit. The number of studies dealing with the objective assessment of the dynamic fit of computer prototypes and their evaluation in terms of comparing the deformations of simulated and real prototypes of clothing models is very limited, given that testing real garments in this sense is very complex and there are no dedicated methods for analysing the deformations of a garment on a moving body in wearing conditions. Pioneering approaches involving 4D scanning and motion capture have introduced methods for overlaying dynamic garment geometries on moving body models [49,50,51]. Klepster et al. developed a clothing fit assessment method during movement, using 4D body scanning. They recorded participants performing movements wearing underwear and conventional upper garments; scans in underwear defined a baseline body shape, and scans in clothing captured possible body restrictions. Using Geomagic Studio 2012 software, overlaying dressed and undressed 4D scans enabled analysis of air gaps and surface penetration, indicating potential restrictions. However, this method is complex, time-consuming, and results depend heavily on participant movement reproducibility, limiting its application to simple movements [49]. Zhang et al. proposed a numerical evaluation method for digital garment prototypes based on surface geometry alignment with 4D-scanned models. They created a simplified kinematic skeleton for animating body surface models, facilitating the overlay of dressed and undressed models. Body motion was defined using point translations and rotations over time, from 4D scans. The undressed model was used to simulate trousers in three movements, which were then overlapped with scans of real trousers during the same movements. Fit was analyzed based on stretch and deformation criteria and overall garment appearance [50]. Terrier developed a FEM-based simulation method for a cycling bodysuit using Abaqus and verified its accuracy using the ARAMIS optical system, which captures 3D deformations commonly used in mechanical and civil engineering and construction. The focus was on analysing compression zones where elastic tapes support the body during cycling [51,52]. Presented methods, though promising, face challenges related to data processing complexity, motion repeatability, and the precise alignment of body and garment geometries. Overall, while current CAD-based garment simulation tools offer significant potential for sustainable, cost-effective, and accurate garment development, the field continues to evolve. Future research should focus on improving dynamic fit modelling, standardizing mechanical property measurement methods, and advancing intelligent systems for fabric property prediction.

In this study, the potential for integrating and defining fabric mechanical parameters within the 3D garment simulation and design environment, CLO3D, was explored to evaluate the influence of different measurement approaches on the results and accuracy of digital clothing prototypes. Mechanical property values for a selected woven fabric sample were determined using three distinct evaluation methods: The Kawabata Evaluation System (KES-FB), the Fabric Kit method, and the artificial intelligence-based application SEDDI Textura 2024. The obtained values were used to define the mechanical behaviour of the fabric during 3D clothing simulation in the CLO3D environment. To validate the resulting digital simulations, the ARAMIS optical 3D measurement system was employed to analyse the dynamic deformation of physical garment prototypes, i.e., base models of a woman’s blouse and trousers. The analysis focused on assessing the degree of accuracy and correlation between simulated and real prototypes’ deformations under dynamic conditions, depending on the method used to derive the fabric parameters. The hypothesis underpinning this work is that fabric properties obtained via AI-assisted evaluation will result in the most realistic simulation outcomes, producing digital prototype deformations that most accurately reflect those observed in physical prototypes subjected to movement. The findings of this study contribute to ongoing efforts to improve the fidelity of 3D garment simulations and to establish best practices for parameterization based on available measurement technologies.

## 2. Materials and Methods

### 2.1. Analysis of Textile Material Properties for 3D Simulation

**Figure 1:**
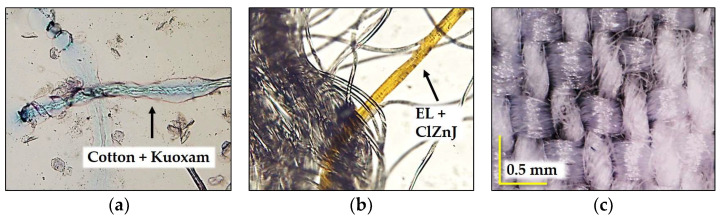
Fiber composition and woven characteristics of selected fabric: (**a**) warp—magnification 100×, cotton + kuoxam = specific swelling effect before dissolution of cotton fibre in kuoxam reagent; (**b**) weft—magnification 100×, elastin + ClZnJ = specific yellowing effect of elastane fibers in ClZnJ reagent; (**c**) fabric surface—magnification 68×, twill weave.

A twill-woven fabric composed of blended fibres, intended for women’s outerwear, was selected for testing. All fabric test samples were pre-conditioned and tested under the standard testing conditions of 20 ± 2 °C temperature and 65 ± 4% relative humidity. The percentage of components is given in Table 1. The fibre composition was qualitatively confirmed through microscopy analysis using specific reagents, employing an Olympus BX51 universal microscope with a DP50 digital camera and image analysis software, AnalySIS 2002. The warp yarn consists of pure cotton, while the weft yarn consists of elastane filaments wrapped with polyester fibres, as shown in Figure 1a,b.

The fabric structure was observed using a Dino-Lite AM7915MZT digital microscope, as shown in Figure 1c. Fabric thread density was determined by counting according to the HRN EN ISO 7211-2:2024 standard [53], with five measurements per sample, expressed as threads per centimeter. Areal weight was measured per HRN ISO 3801:2003 [54], using an analytical balance on three conditioned circular samples (100 cm^2^), and results were recalculated to g/m^2^. Fabric thickness was determined following HRN ISO 5084:2003 [55], using a thickness gauge with a 50 mm diameter presser foot and 1 kPa pressure. Ten individual measurements were taken per sample and reported as mean thickness in millimeters. The structural and physical parameters are summarized and presented in Table 1.

#### 2.1.1. Investigation of Fabric Mechanical Parameters Using the KES-FB System

The mechanical parameters required for 3D garment simulation were determined using the Kawabata Evaluation System (KES-FB) for the objective evaluation of textiles and clothing [2,30]. Since the KES system expresses the values of the determined parameters in SI system units of measurement, for entry into the CLO3D program, the parameter values were recalculated into the measurement units supported by CLO3D and were directly entered and saved as a new material via the Fabric Properties Editor menu [31]. The mechanical parameters obtained using the KES-FB system and recalculated values imported into the CLO3D software are presented in Table 2.

#### 2.1.2. Investigation of Fabric Tensile and Shear Properties Using a Fabric Kit Adapted Method

The fabric was further evaluated using the parameters defined by the Fabric Kit measurement system, designed for CLO3D. Although original Fabric Kit devices were not used, tensile and shear parameters were determined using a tensile testing machine, Tensolab 3000, Mesdan S.p.A. (Raffa, Italy), with testing conditions aligned to Fabric Kit guidelines: a 120 mm grip separation and specimen dimensions of 270 × 30 mm, tested in warp, weft, and bias directions. Force readings were performed during five consecutive extensions of 1 or 10 mm, depending on the elasticity of the fabric. The method prescribes that if a force of less than 0.098 N is required to stretch the material by 1 mm (elongation of 0.83%), the force is recorded at five consecutive elongations of 10 mm each (8.33%), or at each increase in force of 0.196 N. If a force greater than 0.098 N is required to stretch the material by 1 mm, the force is recorded at five consecutive elongations of 1 mm each (0.83%) [10,31]. The determined results of five consecutive readings of the force values required for defined increments of elongation of the test specimen were entered into the Emulator module within the CLO3D software, which converts the entered values into supported units of measurement and creates a compatible data format of the new digital material for application in 3D clothing simulation processes, as shown in Table 3.

#### 2.1.3. Digitization of Textile Material Using the SEDDI Textura AI Application

The SEDDI Textura AI application was used to digitize the selected woven fabric based on scanned surface images and input of basic data: fibre composition, weave type, areal weight, and thickness [31,34]. The method integrates photogrammetry, spectrometry, and computer vision to extract detailed surface geometry and optical reflectance data from scanned images of textile materials. These data, in combination with manually input data on fiber composition and basic physical parameters, are processed through a deep learning algorithm that trains a neural network to generate parameters suitable for physically based rendering. A key advantage of the AI method lies in its use of a multimodal dataset that combines visual, spectrometric, and mechanical properties of textiles, thereby capturing a broad spectrum of real-world material variations. The neural network is trained on a large number of samples with diverse textures, thicknesses, fiber compositions, and mechanical characteristics. This enables the model to generalize fabric behavior beyond the original dataset. The final result is a digital texture ready for direct integration into 3D CAD software, containing data for realistic visual representation and assessed values of material mechanical parameters for 3D garment simulation [31,34]. The accessed values of the mechanical parameters suitable for CLO3D software and selected woven fabrics are presented in Table 4.

### 2.2. Development of Digital 3D Prototypes and Static Fit Analysis

**Figure 2:**
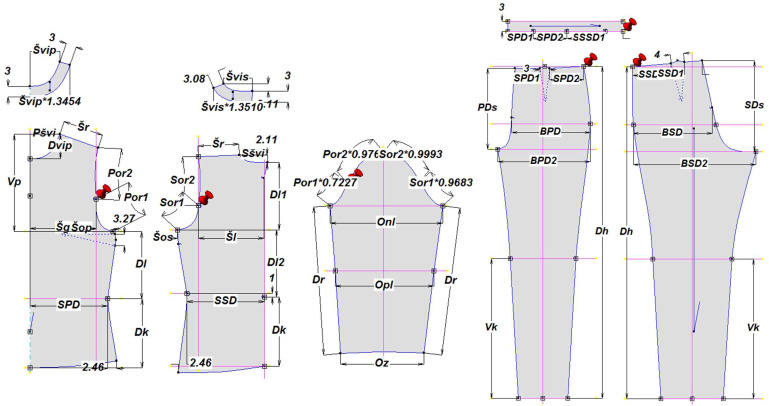
Parametric cutting patterns of the women’s blouse and trousers adjusted according to the anthropometric characteristics of the test subjects’ scanned 3D models. Legend: Pšvi—front neckline width; Švip—front neckline width on hem lining; Šr—shoulder width; Dvip—front neckline depth; Vp—front height; Šg—bust width; Šop—front armhole width; Por1—front armhole curve length 1; Por2—front armhole curve length 2; SPD—front waist width; Dl—back length; Dk—pattern length; Sšvi—back neckline width; Švis—back neckline width on hem lining; Šl—back width; Šos—back armhole width; Sor1—back armhole curve length 1; Sor2—back armhole curve length 2; SSD—back waist width; Dr—sleeve length; Onl—upper arm girth; Opl—lower arm gith; Oz—wrist girth; SPD1—front waist width 1; SPD2—front waist width 2; SSD1—back waist width 1; SSD2—back waist width 2; BPD—front hips width; BSD—back hips width; BPD2—front hips width at crotch level; BSD2—back hips width at crotch level; PD_s_—front crotch heigth; SD_s_—back crotch heigth; Dh—trousers length; Vk—knee heigth.

The basic women’s blouse and trouser models were selected for the investigation, in order to eliminate the influence of design and cutting pattern complexity on the fit analysis and to focus solely on evaluating the simulation process and the impact of fabric mechanical parameters on the prediction of garment appearance and behavior in both static and dynamic conditions. Parametric cutting patterns that enable automatic adjustment of pattern segments according to defined interdependencies between segments’ dimensions and targeted anthropometric characteristics were developed using the Modulate program, Optitex, as shown in Figure 2 [56,57]. The patterns were customized based on the body measurements of a selected test subject obtained by 3D body scanning.

**Figure 3:**
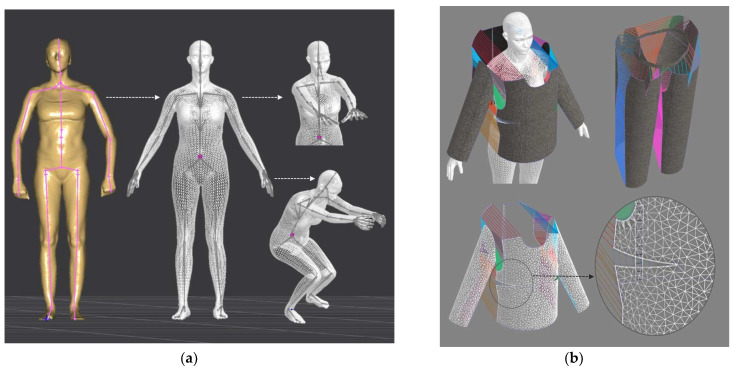
Development of digital 3D prototypes: (**a**) development of custom animated kinematic avatar for 3D simulation based on scanned 3D body model of the test subject; (**b**) simulation parameters including joining segments and surface mesh density.

**Figure 4:**
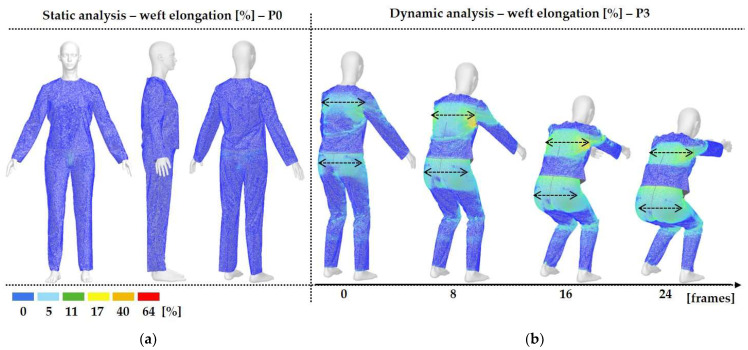
Fit analysis of women’s blouse and trousers digital prototypes simulated based on SEDDI Textura parameters: (**a**) weft elongation in static upright position P0; (**b**) monitoring weft elongation throughout the complete sequence of P3 movement.

Digital 3D prototypes of basic models were developed using the CLO3D software. An animated kinematic avatar, developed based on the test subject’s scanned 3D model, was used for simulations and analysis of the garment on the body in motion, as shown in Figure 3a. All necessary simulation parameters, including component positioning, joining segments and surface mesh resolution, i.e., polygon size set to 5 mm were defined, as shown in Figure 3b, and the simulations involving application of each set of mechanical parameters from the three evaluation methods were performed. The digital prototypes were first analyzed in a static standing position, confirming dimensional alignment of the pattern and underlying body model, with expected ease allowance across bust, waist, and hip circumferences (1–4 cm). The deformation analysis of the digital prototypes was performed in the directions corresponding to the warp and weft of the simulated woven fabric using the Strain map measurement tool. The strain map tool allows analysis of how much clothing stretches during wear in a particular direction, allowing the estimation of deformations when transforming surface 2D cutting parts (flat, undeformed fabric surface) into the 3D garment form on the avatar’s body. All prototypes, regardless of the applied mechanical property parameters, presented 0% fabric elongation in the standing upright position (P0), further confirming good dimensional fit of the prototypes in static conditions (Figure 4a).

### 2.3. Surface Deformations Analysis of Digital Prototypes in Dynamic Conditions

The digital prototypes, developed using three different sets of mechanical parameters, were further analysed on an animated avatar in three body movements: horizontal arm abduction, forward arm flexion, and squatting. The avatar is animated according to precisely defined kinematic chains of given movements, which determine the types of translation and rotation in the relevant joints, to achieve the targeted final position, as well as the rhythm and speed of movement execution. Surface deformations, caused by contact mechanics between the avatar in motion and digital garments, and based on the input set of fabric mechanical parameters, were monitored throughout the complete sequences of the given movements, as shown in Figure 4b. Transverse and longitudinal deformations, corresponding to warp and weft directions, were analyzed at the relevant garment zones.

**Figure 5:**
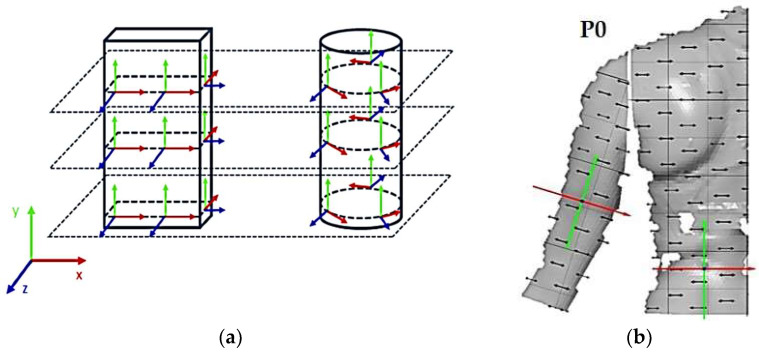
Presentation of the local coordinate system on the surface of a recorded object in the Aramis system; (**a**) General schematic representation; (**b**) Adjustment on a recorded human body model segment.

On the blouse model prototype, the upper back torso area was analyzed during arm movement, in the transverse direction corresponding to the weft direction of the simulated fabric. The trousers model prototypes were analyzed during a squatting movement, in both transverse (weft) direction on the hip area and longitudinal (warp) direction, observed on the back of the body in the hip and thigh areas (Figure 5), as well as on the front knee area. At the determined zones of maximum deformation in a particular movement, exact deformation values were measured at 10 measurement points, randomly selected within the zone of maximum deformation, and the results were expressed as the mean value of individual measurements.

### 2.4. Digital Prototypes Evaluation Based on the Applied Set of Mechanical Parameters

For the purpose of verifying digital 3D prototypes and analyzing deformations under dynamic conditions, physical prototypes of a women’s blouse and trousers were created, tailored to the anthropometric characteristics of the target subject. The prototypes were recorded under real wearing conditions, i.e., during the execution of predefined movements, using the optical 3D measurement system known as Aramis for dynamic deformation analysis. The Aramis system, GOM GmbH, Germany, is an optical system for 3D deformation analysis based on the stereophotogrammetry method, in which three-dimensional deformations of the recorded object are reconstructed from two or more images taken from different angles. The measurement methodology involves the test model preparation by creating a high-contrast stochastic dot pattern, which serves as a reference for determining point coordinates and tracking displacements and deformations on the surface of the object in motion. The initial global coordinate system is usually defined at the first calibration image by the position of the calibration panel. The stresses in the *x*-direction are always calculated as material coordinates, i.e., as local coordinates that move with the material [47]. Therefore, each point has its own coordinate system, as shown in Figure 5.

The program calculates the stress in the moving coordinate system, not the global coordinate system. The *z*-direction is used as the thickness direction. The program uses the normal compensation plane around the corresponding point in the direction. The local *x*-direction results as the product of the intersection of the normal plane vector and the global *y*-axis, and the local *y*-direction results as the product of the local *z* and *x*-axes [47]. Stress is defined as the ratio of the elongation to the reference initial length due to the action of a force. In technical stress, the elongation ratio of elements is the quotient of the current length l_1_ and the reference length l_0_ and can be represented by the expression (1):

(1) $$\Lambda =\frac{l_{1}}{l_{0}}=\frac{l_{0}+\Delta l}{l_{0}}=1+\varepsilon \Leftrightarrow l_{1}=l_{0}\Lambda =l_{0}(1+\varepsilon )\Rightarrow \Lambda =1+\varepsilon$$

Deformations of a body can be considered in several steps. If a body is elongated by Δl at each deformation step, the body has a new reference length, which means that the total stress is not equal to the sum of the individual stresses. The infinitesimal change in stress of an element is defined as (2):

(2) $$d\varepsilon^{\prime }=\frac{dl}{l}$$

Therefore, stress can be viewed as the integral of an infinitesimal change (3):

(3) $$\varepsilon^{\prime }=\int_{l_{0}}^{l_{1}}\frac{1}{l}dl={\left[\ln\left(l\right)\right]}_{l_{0}}^{l_{1}}=\ln\left(l_{1}\right)-\ln\left(l_{0}\right)=\ln\left(\frac{l_{1}}{l_{0}}\right)=\ln\left(\Lambda \right)=\ln\left(1+\varepsilon \right)$$

**Figure 6:**
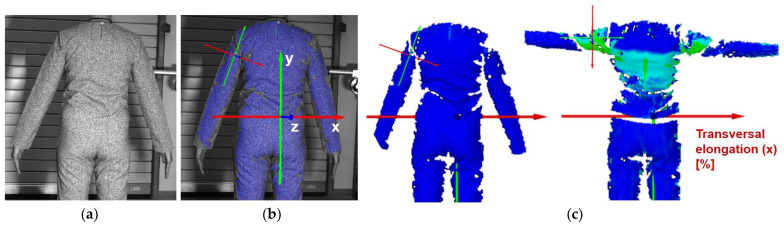
Analysis of women’s blouse prototype in real wearing conditions: (**a**) Real clothing model prototype; (**b**) Creation of measuring surfaces and adjustment of the segments coordinate system; (**c**) Transverse (x) deformations analysis in the initial position P0 and the position P1.

**Figure 7:**
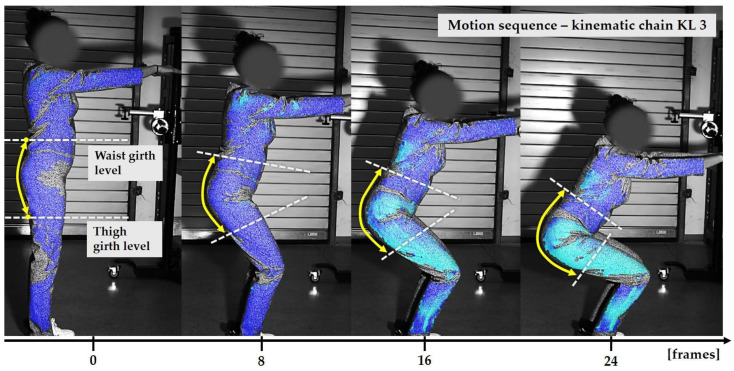
Monitoring of longitudinal (y) deformations of women’s pants prototypes during squatting movement.

For the purpose of the study, a stochastic dot pattern was applied on selected woven fabric using digital printing technology prior to the experimental part of the research. The ZEISS Inspect software v.2023, used for processing and analyzing the recorded data, offers a wide range of tools to extract deformation metrics that are comparable to the parameters used in the analysis of 3D simulations and digital garment prototypes. The processing of the recorded Aramis data included the creation and segmentation of measurement surfaces and the adjustment of the coordinate system of each surface segment according to the warp and weft directions of the particular garment part (Figure 6b). Physical prototypes of the blouse and trousers (Figure 6a) were analyzed during the three movements, in the transversal (Figure 6c), and longitudinal directions (Figure 7). In the determined zones of maximum deformation of the garment in a particular movement, analogous to the method applied in the analysis of digital prototypes, the elongation values were measured for 10 randomly selected points within the zone of maximum deformation, and the results were expressed as the mean value of individual measurements. Comparative analysis of geometric deformations between physical and simulated prototypes was conducted, and the outcomes of the 3D simulations, performed using sets of mechanical parameters determined by three different characterization methods, were evaluated.

## 3. Results and Discussion

Comparative analysis of the observed deformations between real and digital prototypes revealed similar deformation zones, up to 6% average differences between simulated and real prototypes with 8% standard deviations, depending on the applied set of mechanical parameters, as shown in Table 5, Table 6, Table 7 and Table 8. Descriptive statistical analysis and arithmetic means of measured garment elongation values according to the determined maximum deformation zones in each movement are shown in Table 7.

Digital prototypes simulated using mechanical parameters determined by the KES-FB system showed higher deformation values compared to the results of the real prototypes analysis. In contrast, simulations using parameters obtained using the Fabric Kit methodology showed lower deformations compared to the real prototypes, as shown in Table 5, Table 6, Table 7 and Table 8. When comparing the KES-FB and Fabric Kit methodologies, the main difference lies in the value of the maximum force applied during the analysis. In the testing of tensile and shear properties, the maximum force applied by the KES system is 490.35 N/m. However, in the Fabric Kit method, by tracking five consecutive elongations of 1 or 10 mm (depending on the test sample), higher forces are recorded compared to the maximum force used in the KES-FB system. Due to the limited maximum force, it is not possible to extract force values for five consecutive elongations of 1 or 10 mm from the F/ε diagram, and parameters obtained from the KES-FB system cannot be imported into the CLO3D software using the Emulator.

When observing the converted parameter values imported in CLO3D, as shown in Table 2 and Table 3, it is evident that the stress values derived from the KES-FB parameters are lower compared to the stresses converted from the parameters obtained using the Fabric Kit method and the SEDDI Textura application. Furthermore, in the direct conversion of tensile and shear parameters obtained from the KES-FB system to CLO3D compatible units, a single elongation value determined at the maximum force is used, meaning the calculated stress parameter is based on just one value. In contrast, the Fabric Kit method, via the Emulator converter, utilizes five consecutive force readings at a defined elongation value, which, when examined in the F/ε diagram, represents a significantly broader testing range.

Digital prototypes, simulated based on SEDDI Textura mechanical properties, showed the closest match to real prototypes in both value and distribution of deformations, as shown in Table 5 and Table 6. The advantage of AI-driven methods like SEDDI Textura is that they are deep learning models trained on large textile datasets, generalizing behavior beyond isolated measurements and capturing nonlinearities in structure/behavior relationships based on data patterns. These systems estimate parameter distributions rather than single values, increasing robustness to variability. Unlike standard physical testing methods, SEDDI Textura employs a multimodal approach that combines visual (2D fabric images) and structural inputs (weave type, fiber content, mass, and thickness), enhancing the precision of deformation predictions.

A pronounced symmetry of deformations was observed on the left and right sides of all digital 3D prototypes. In contrast, the real prototypes showed differences in the deformation’s distribution on the left and right sides. The observed limitation is a consequence of the computer 3D body model symmetry, as well as the symmetry of the kinematic transition of joints in the animation sequences.

**Figure 8:**
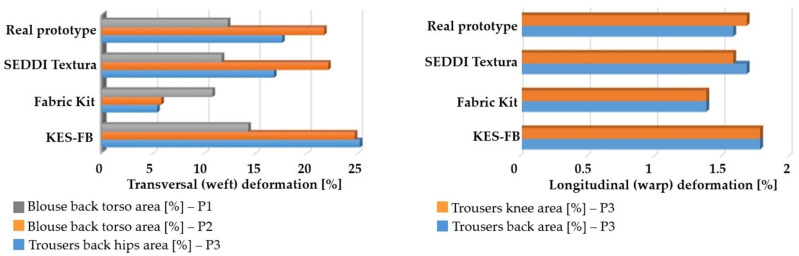
Comparison of fabric elongation values observed in dynamic motion conditions between the digital prototypes simulated using three different characterization methods of mechanical parameters and validation with real prototypes.

Figure 8 presents a comparison of the determined elongation values for the blouse and trouser prototypes observed in different movement positions (P1, P2, and P3), between the simulation results derived using three different fabric characterization methods and tested real garment prototypes using the ARAMIS 3D optical system. The graphs highlight consistent deviations in deformation values that can be attributed to the different methodologies used for parameter characterization.

The KES-FB system systematically predicts higher deformation values, as shown in Table 7, due to the low load testing and prediction of behavior related to lower stiffness parameter values. Maximum force used by the KES-FB System (490.35 N/m) ensures that the testing is limited to the linearity range, which possibly does not reach a sufficiently wide range of deformations required for CAD simulation, especially for materials with higher elasticity. The Fabric Kit method predicts lower deformations, as shown in Table 7, likely due to the high force values and limited sensitivity to low-strain responses, resulting in stiffer behavior in the simulation environment. The SEDDI Textura method consistently shows the lowest deviation in relation to the test results of real prototypes, as shown in Table 8, with the most accurate distribution by tested zones, which indicates the highest predictive accuracy in dynamic conditions.

**Figure 9:**
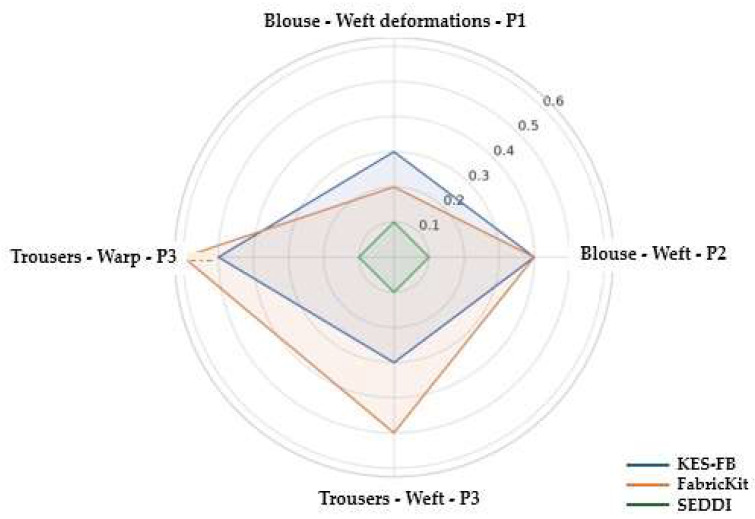
Radial graph of absolute deformation deviations between simulated and real prototypes for three different characterization methods of mechanical properties.

Figure 9 illustrates the absolute differences in fabric deformation between digital and real prototypes, tested in specific motions. The axes represent deformations of clothing models in weft and warp directions, while the radial distance reflects the deviation in value. The obtained results support the hypothesis that digitalization of fabric using AI technology will yield realistic simulation results, achieving deformations of digital prototypes that accurately reflect the real behavior of the body in motion, highlighting the potential of machine learning tools in improving the accuracy and sustainability of digital clothing prototypes.

The overall results of the digital prototype evaluation revealed significant data on the advantages and limitations of the compared methods for characterizing fabric properties, systematically presented in Table 9.

The significance of the presented study lies in its integrative and comparative approach to evaluating the influence of different methods for determining fabric mechanical properties on the accuracy of 3D garment simulations under dynamic conditions. While numerous previous studies have investigated the fidelity of garment simulations using subjective visual assessments [41,42], cross-sectional comparisons [48], or static 3D scanning methods [40,47], relatively few have focused on validating digital garment behavior through objective, quantitative analysis of deformation during motion. In contrast to studies that evaluate static drape accuracy or rely primarily on visual similarity [40,41,42,43], our research employs the ARAMIS optical 3D measurement system to capture and compare the dynamic deformations of physical and virtual garment prototypes. This methodological advancement enables a more precise and objective evaluation of simulation outcomes under realistic wearing conditions, addressing a major limitation identified in earlier research [49,50,51].

By directly comparing three different methods for fabric parameter acquisition, KES-FB, Fabric Kit, and the AI-based SEDDI Textura application, within the same simulation environment (CLO3D), using identical garment models, textile material and human test subject, allows for a controlled analysis of how the source and quality of mechanical input data influence the resulting simulation fidelity. Notably, the findings demonstrate the potential of AI-assisted tools to generate accurate garment deformations during movement. Confirming the effectiveness of SEDDI Textura in predicting mechanical properties for 3D clothing simulation supports the advancement of sustainable, data-driven workflows in digital garment development [31,32,33,34].

## 4. Conclusions

The conducted research confirmed the feasibility of employing various methods for determining the mechanical properties of woven fabrics for digital 3D garment simulations under dynamic conditions. The integration of KES-FB, Fabric Kit, and AI-based SEDDI Textura into the CLO3D environment enabled realistic prediction of fabric deformation, which was validated through physical garment prototypes and 3D motion analysis. The ability to objectively evaluate physical prototypes in dynamic conditions, as demonstrated using the ARAMIS system, provides a reliable framework for assessing digital simulation outcomes and offers significant insights into the influence of mechanical parameters on 3D simulation accuracy, depending on the testing methods and protocols adopted by each measurement system.

The scientific contribution of this study lies in the quantitative comparison between simulated and actual garment behavior using an optical 3D measurement system for dynamic deformation, combined with advanced fabric characterization techniques. A comprehensive comparative analysis was performed between the deformation of digital and real garment prototypes under identical dynamic conditions, using the same garment models and materials. Methodologically, the study demonstrated the effectiveness of using the ARAMIS system for the quantitative verification of digital prototypes in the context of garment design, with allowance for ease, paving the way for its broader application in apparel engineering.

The study also highlights the potential of AI-based tools in replacing traditional testing setups with sustainable digital approaches. The validity of using AI technology (SEDDI Textura) to estimate mechanical parameters of textile materials, based solely on structural data and visual appearance, was experimentally confirmed, contributing to the advancement of sustainable digitalization methods without the need for conventional testing. The advantage of the SEDDI method stems from its combination of scalability, learning from large datasets, multimodal input, and optimization towards simulation outcomes rather than isolated measurements. Consequently, the simulation results generated using its predictions exhibit higher correspondence with the behavior of real garments, affirming its potential value for sustainable and industry-relevant apparel design.

The implications of the presented research findings can be observed from the practical, social and managerial aspects of application. This research confirms that the application of digital prototyping with the support of AI-based tools, such as SEDDI Texture, can significantly reduce the number of physical prototypes and the amount of textile waste, thereby reducing product development time and optimizing costs. The practical benefit for designers and manufacturers is an increase in the accuracy of material simulation, a reduction in material and transportation costs, and a faster response to changes in market demand. A particularly valuable contribution of this research is the introduction of a new methodology for the analysis and evaluation of prototypes using the ARAMIS optical 3D measurement system, which enables accurate measurement of fabric deformations and displacements. In this way, the results of digital simulation can be verified by real measurements, which ensures a credible interpretation of the results of digital analysis and increases the reliability of digital prototypes in industrial applications. The results indicate that the digital transformation of the textile and fashion industry has wider societal benefits through the reduction of waste, CO_2_ emissions and negative environmental impact. However, this transformation also opens up new challenges and opportunities in the field of employment and education.

New educational programs and curricula need to be developed that will enable young professionals and the existing workforce to acquire specialized digital skills, such as 3D modeling, working with CAD/CAM systems, digital prototyping, data management, and working with AI-based tools. This requires collaboration between industry, academia, and public policies in developing lifelong learning programs and adapting vocational education to the needs of Industry 4.0. In this way, the digital transition contributes not only to a more sustainable industry but also to strengthening employability, developing local communities and retaining talent. The findings indicate the need for managers in the textile sector to strategically plan investments in digital tools, staff training and adaptation of internal processes for the implementation of Industry 4.0. Organizations that adopt digital prototyping can gain a competitive advantage through faster innovation, resource optimization and more transparent supply chains.

Future research should be extended to include various fabric structures and garment types, with added focus on modeling textile finishing processes and refining AI-based predictive systems through deep learning algorithms and feedback from experimental results. The scientific insights gained from this study represent a meaningful step towards a more sustainable and efficient apparel development process and encourage further advancement of intelligent methods for virtual prototyping based on objective, digital, and automated approaches to material characterization.

## Figures and Tables

**Table 1:** Structural and physical parameters of selected woven fabric M1 for digital prototyping.

Woven Fabric M1	Fibre Composition	Wave Type	Density	Areal Weight	Thickness
			[Threads per cm]	[g/m^2^]	[mm]
Warp	65% cotton 33% polyester (PES) 2% elastane (EL)	Twill	40	226.58	0.52
Weft			20		

**Table 2:** Values of mechanical parameters determined by the KES-FB system.

Tensile Parameters		Bending Parameters		Shear Parameter
EMT-1 [%] *	EMT-2 [%] *	B-1 [cNcm] *	B-2 [cNcm] *	G [cN/(cm°)^−1^] *
1.730	11.180	0.356	0.093	0.010
Stretch Warp [g/s^2^]	Stretch Weft [g/s^2^]	Bend Warp [gmm^2^/s^2^]	Bend Warp [gmm^2^/s^2^]	Shear [g/s^2^]
482,012	441,042	3560	930	100,000

* EMT-1—warp elongation at 490.35 cN load; EMT-2—weft elongation at 490.35 cN load; B-1—warp bending stiffness; B-2—weft bending stiffness; G—shear stiffness.

**Table 3:** Determination of tensile and shear parameter values using the adapted Fabric Kit method.

Parameter	Warp	Weft	45° Direction
Extension Interval [mm]	1	10	10
Elongation Interval [%]	0.83	8.33	8.33
1	3.1	3.3	2.7
2	8.0	10.8	5.1
3	17.7	47.1	9.7
4	29.8	139.8	20.6
5	45.5	214.9	45.9
CLO3D convert [g/s^2^]	1,000,000	590,885	436,596

**Table 4:** Values of mechanical parameters determined by the SEDDI Textura AI application.

Stretch-Warp	Stretch-Weft	Bend-Warp	Bending-Weft	Shear Rigidity	Areal Weight
[g/s^2^]	[g/s^2^]	[gmm^2^/s^2^]	[cNcm]	[g/s^2^]	[g/m^2^]
691,472	425,985	4651	2578	89,210	226

**Table 5:** Results of the transversal (weft) deformations analysis of real and digital 3D prototypes of a woman’s blouse in movements P1 and P2 from the aspect of the applied set of mechanical parameters.

	Transversal (Weft) Elongation [%]	
Position	P1	P2
KES-FB	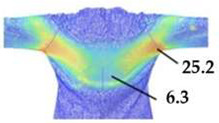	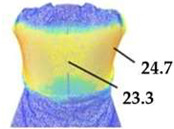
Fabric Kit	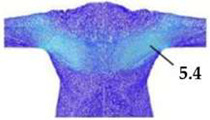	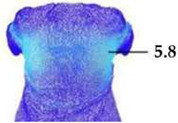
SEDDI	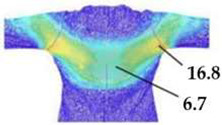	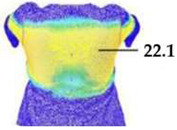
Real prototype	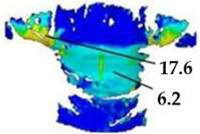	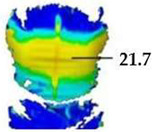
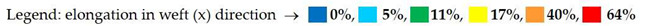		

**Table 6:** Results of the longitudinal (warp) and transversal (weft) deformations analysis of women’s trousers, real and digital 3D prototypes in movement P3, from the aspect of the applied set of mechanical parameters.

	Transversal (Weft) Elongation [%]	Longitudinal (Warp) Elongation [%]	
Position	P3—Posterior	P3—Frontal	P3—Sagittal
KES-FB	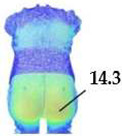	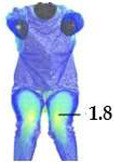	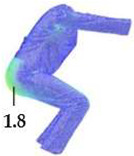
Fabric Kit	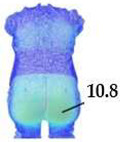	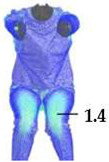	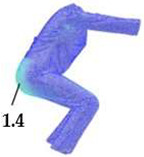
SEDDI	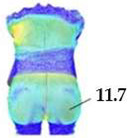	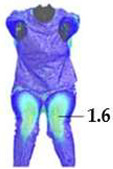	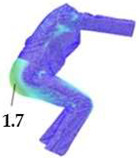
Real prototype	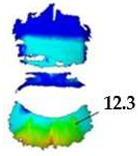	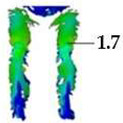	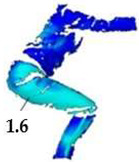
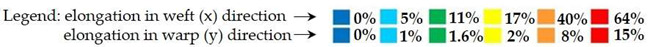			

**Table 7:** Descriptive statistical parameters of determined elongation values of digital and real clothing prototypes in observed maximum deformation zones.

Movement/Position	Observed Garment Zone		Digital Prototypes			Real Prototype
			KES-FB	Fabric Kit	SEDDI Textura	Aramis
P1	Upper back torso (x/weft direction)	Mean strain [%]	25.22	5.43	16.79	17.62
		Standard deviation	0.91	0.43	0.42	0.25
		Min.	24.1	4.8	16.1	17.3
		Max.	27.1	6.1	17.5	18.2
P2	Upper back Torso (x/weft direction)	Mean strain [%]	24.71	5.79	22.12	21.71
		Standard deviation	0.65	0.44	0.34	0.17
		Min.	23.5	5.1	21.6	21.4
		Max.	25.4	6.3	22.5	22.0
P3	Back hips (x/weft direction)	Mean strain [%]	14.28	10.82	11.7	12.31
		Standard deviation	0.65	0.43	0.44	0.18
		Min.	13.5	10.1	10.9	12.0
		Max.	15.5	11.4	12.3	12.6
	Front knee (y/warp direction)	Mean strain [%]	1.81	1.41	1.62	1.69
		Standard deviation	0.18	0.13	0.21	0.10
		Min.	1.5	1.2	1.3	1.5
		Max.	2.1	1.6	1.9	1.8
	Back length, waist to thigh (y/warp direction)	Mean strain [%]	1.80	1.44	1.70	1.61
		Standard deviation	0.16	0.19	0.26	0.13
		Min.	1.5	1.2	1.3	1.4
		Max.	2.1	1.8	2.1	1.8

**Table 8:** Descriptive statistical parameters of determined differences in elongation values of digital and real clothing prototypes in observed maximum deformation zones.

Position	Observed Garment Zone	$\mathrm{Absolute}\;\mathrm{Differences}\;\left|∆\right|$		
		KES-FB	Fabric Kit	SEDDI Textura
P1	Upper back torso (x/weft direction)	7.6	12.2	0.8
P2	Upper back torso (x/weft direction)	3.0	15.9	0.4
P3	Back hips (x/weft direction)	2.0	1.5	0.6
	Front knee (y/warp direction)	0.1	0.3	0.1
	Back length waist to thigh (y/warp direction)	0.2	0.2	0.1
Mean differences value [%]		2.58	6.02	0.40
	Standard deviation	3.06	7.46	0.31
	Min.	0.1	0.2	0.1
	Max.	7.6	15.9	0.8

**Table 9:** Comparison of fabric characterization methods for 3D clothing simulation.

**Method**	**Technical Description**	**Advantages**	**Limitations**	**Integration with CLO3D**
KES-FB	Highly sensitive laboratory system for testing mechanical properties under small loads.	High measurement precision. Detailed quantification of mechanical parameters. Good simulation results.	Limited standard maximum force. One measurement data entry. Incompatibility with CAD formats. Expensive equipment.	Low Required conversion
FabricKit	Simple measuring system with defined protocol, compatible with Emulator converter for CAD import.	Quick and simple process. Good simulation results. Direct integration in CLO3D. Optimal possibilities for design applications.	Less sensitive to fine deformations. Five measurement data entry. High testing range, which in some cases exceeds the linearity limit.	High Integrated conversion
SEDDI Textura	AI tool for prediction of textile mechanical parameters based on 2D image and basic data.	No physical testing. Fast implementation. Multimodal approach. Good prediction results. Sustainable methodology.	Sensitivity to the quality of input data. Require further experimental validation.	High Direct data input

## Data Availability

Data are contained within the article.
